# Pemetrexed pharmacokinetics and pharmacodynamics in a phase I/II study of doublet chemotherapy with vinorelbine: implications for further optimisation of pemetrexed schedules

**DOI:** 10.1038/sj.bjc.6603995

**Published:** 2007-10-02

**Authors:** K M Li, L P Rivory, S J Clarke

**Affiliations:** 1 Discipline of Pharmacology, Faculty of Medicine, Bosch Institute, School of Medical Sciences, University of Sydney, Sydney, New South Wales 2006, Australia; 2 Johnson and Johnson Research Pty. Ltd, Strawberry Hills, New South Wales 2012, Australia; 3 Department of Medicine, Concord Hospital, Concord, New South Wales 2137, Australia

**Keywords:** thymidylate synthase inhibition, pemetrexed, anti-folates, pharmacodynamic markers, plasma deoxynucleosides, homocysteine

## Abstract

The purpose of this study was to investigate the utility of plasma pharmacokinetic and pharmacodynamic measures including plasma deoxynucleosides, homocysteine and methylmalonic acid concentrations in understanding the time course and extent of the inhibition of thymidylate synthase (TS) by pemetrexed in the context of a phase I/II combination study with vinorelbine. Eighteen patients received supplementation with folic acid and Vitamin B_12_ 1 week before beginning treatment with pemetrexed and vinorelbine administered in a dose-escalating manner on a 21-day cycle. Heparinised blood samples were collected from consenting patients in the first cycle for pharmacokinetic analyses and in the first two cycles for determination of plasma thymidine, deoxyuridine, homocysteine and methylmalonic acid concentrations. These values were correlated with response and toxicity. Plasma deoxyuridine was used as a measure of TS inhibition, and concentrations of deoxyuridine were significantly elevated relative to baseline on days 1 (*P*<0.01), 2 (*P*<0.001) and 3 (*P*<0.05) after treatment at all pemetrexed dose levels (400–700 mg m^−2^). The magnitude of deoxyuridine elevation correlated with pemetrexed area under the plasma concentration–time curve (AUC) (*r*^2^=0.23, *P*<0.05). However, deoxyuridine concentrations returned to baseline between 8 and 15 days after treatment with pemetrexed, suggesting that inhibition of TS was not durable. Pemetrexed AUC correlated with the percentage decline (relative to baseline) in both platelets (*r*^2^=0.58, *P*<0.001) and leucocytes (*r*^2^=0.26, *P*<0.05) at day 8. Baseline homocysteine was also significantly correlated with these measures of haematological toxicity (*r*^2^=0.37, *P*<0.01 and *r*^2^=0.39, *P*<0.01, respectively). In addition, there was a significant reduction of plasma homocysteine on days 8 (*P*<0.005) and 15 (*P*<0.05) in cycle 1 compared to baseline values. The results suggest that the TS inhibitory effects of pemetrexed are short-lived and make the case for a more frequent schedule of administration such as every 2 weeks. The lack of protracted TS inhibition may be due to concomitant vitamin administration, and this may be the mechanism by which vitamins prevent life-threatening toxicity from pemetrexed. Baseline homocysteine concentration remains a predictive marker for haematological toxicity even following folate supplementation.

Pemetrexed, a multitargeted anti-folate, has shown activity in various tumours, especially mesothelioma and non-small-cell lung cancer (NSCLC) for which it is routinely used (Adjei, 2003). Pemetrexed inhibits at least three of the enzymes involved in folate metabolism, and pyrimidine and purine biosynthesis, as shown in Figure 1. These include thymidylate synthase (TS), dihydrofolate reductase (DHFR) and glycinamide ribonucleotide formyltransferase. However, inhibition of TS has been shown to be the primary mechanism of action of pemetrexed (Grindey *et al*, 1992). TS, an *N*^5^,*N*^10^-methylene tetrahydrofolate (methylene-THF)-dependent enzyme, catalyses the reductive methylation of deoxyuridylate (dUMP) to deoxythymidylate, the rate-limiting step in the *de novo* synthesis of deoxythymidine triphosphate. Inhibition of DHFR by pemetrexed reduces the regeneration of the essential coenzyme methylene-THF thereby further enhancing the TS inhibitory effects.

Inhibition of TS in tissues leads to intracellular accumulation of dUMP and subsequent efflux of deoxyuridine (dUrd) into the circulation. Accumulation of dUMP occurs as a direct consequence of TS inhibition and also as a result of increased activity of other enzymes, including ribonucleotide reductase (RR) and deoxycytidylate (dCMP) deaminase (Maybaum *et al*, 1981). A number of studies have indicated that plasma dUrd is an important pharmacodynamic (PD) marker of TS inhibition (Mitchell *et al*, 1997; de Jonge *et al*, 2002; Ford *et al*, 2002; Rees *et al*, 2003) especially with the use of the TS inhibitors AG337 (Nolatrexed) (Jodrell *et al*, 1999; Estlin *et al*, 2001), ZD9331 (de Jonge *et al*, 2002; Ford *et al*, 2002; Rees *et al*, 2003) and ZD1964 (raltitrexed) (Ford *et al*, 2002; Horton *et al*, 2005); however, similar studies have not been performed with pemetrexed.

Inhibition of TS may be offset by thymidine (TdR) salvage pathways, and administration of exogenous TdR has been shown to rescue tumour cell lines from pemetrexed-induced cytotoxicity (Smith *et al*, 1999). Therefore, basal plasma TdR concentrations might be predictive for response and toxicity with TS inhibitors. However, correlative assessments of TdR and toxicity have not previously been possible because of the lack of an appropriately sensitive and specific assay. We have recently developed and validated a liquid chromatography mass spectrometry (LC-MS) assay that has demonstrated that human plasma TdR concentrations are much lower than previously reported (Li *et al*, 2003).

Myelosuppression is the principal toxicity following treatment with pemetrexed with 50% of patients experiencing grade 3/4 toxicity (Hanauske *et al*, 2001). Early in the preclinical evaluation of pemetrexed, it became apparent that functional folate status was an important factor in the activity and toxicity of this compound (Worzalla *et al*, 1998). Methionine synthase, the enzyme which catalyses the conversion of homocysteine (Hcy) to methionine (Met), requires *N*^5^-methyltetrafolate (methyl-THF) as a methyl group donor and vitamin B_12_ for this reaction to proceed. When methyl-THF and vitamin B_12_ levels are low, the conversion of Hcy to Met decreases, resulting in increased concentrations of Hcy (Figure 1). Thus, Hcy can be used as a marker for overall folate status in the body. Likewise, vitamin B_12_ is a vital cofactor for methylmalonyl coenzyme A mutase, and the level of methylmalonyl acid (MMA) increases as a result of vitamin B_12_ deficiency (Savage *et al*, 1994; Lucock *et al*, 1999). It has been shown that plasma Hcy and MMA levels are important markers in predicting severe toxicity with pemetrexed (Niyikiza *et al*, 2002).

Administration of folic acid (0.5 mg per mouth each day) and vitamin B_12_ (1 mg by intramuscular injection every 9 weeks) commencing 1 week prior to treatment with pemetrexed has been shown to reduce the incidence of life-threatening toxicity, especially myelosuppression (Bunn *et al*, 2001). However, the use of vitamin prophylaxis permits higher doses of pemetrexed to be safely used, creating some uncertainty about the optimal schedule and dose of this agent in the post-vitamin era. Consequently, a comparison of the clinical pharmacokinetics (PK) of pemetrexed with markers of its PD effects could clarify these issues of optimal dose and schedule.

Previously, we have reported that the combination of pemetrexed and vinorelbine is well tolerated and shows activity as first-line treatment in advanced NSCLC patients (Clarke *et al*, 2005). The objective of the current study was to further investigate the PD of TS inhibitory effects by analysing plasma dUrd and TdR concentrations and correlating them with toxicity in these patients. By including a PK analysis of pemetrexed, another objective was to assess possible PK–PD correlations during the first course of treatment. Furthermore, we wished to correlate drug-induced toxicities with functional folate status in vitamin-supplemented patients.

## MATERIALS AND METHODS

Eighteen patients from the phase I/II study of pemetrexed and vinorelbine at the Sydney Cancer Centre at Royal Prince Alfred Hospital agreed to participate in this substudy. The demographics and clinical characteristics of the patients are summarised in Table 1. Eligibility criteria for patient recruitment were NSCLC with at least aged 18 years with minimum estimated 12 weeks life expectancy and performance status 0–2; good bone marrow reserve (platelets>100 × 10^9^ l^−1^, haemoglobin>90 g l^−1^, absolute granulocyte count>2.0 × 10^9^ l^−1^); adequate hepatic function (bilirubin<1.5 × upper limit of normal (ULN), transaminase<3.0 × ULN (unless liver metastases present)); and adequate renal function (creatinine clearance>0.75 ml s^−1^, albumin>25 g l^−1^). Exclusion criteria were symptomatic brain metastases, uncontrolled pleural or peritoneal effusions, and inability to interrupt non-steroidal anti-inflammatory drugs. The study was approved by the Human Ethics Research Committees of the Central Sydney Area Health Service and the University of Sydney.

### Treatment

The patients received 400–700 mg m^−2^ pemetrexed (Alimta^©^, Eli Lilly and Company, Indianapolis, IN, USA) as a 10-min infusion on day 1 of a 21-day cycle. Vinorelbine was infused at 15–30 mg m^−2^ (approximately 6–10 min infusion) on days 1 and 8 of a 21-day cycle. On day 1, the vinorelbine infusion began 30 min after the end of the pemetrexed infusion. This 21-day period defined one cycle of therapy. All patients received supplementation with folic acid (500 *μ*g p.o., daily) and vitamin B_12_ (1000 *μ*g i.m., every 9 weeks) commencing 1 week before the first cycle and continuing until the discontinuation of chemotherapy.

### Blood samples collection procedure

For the PK study, heparinised blood samples were collected at time 0 (baseline), 9.5 min (end of pemetrexed infusion), 15, 30, 50 min (end of vinorelbine infusion), 1 h, 1 h 20 min, 2, 4, 8, 24, 48 and 72 h, for measurement of plasma pemetrexed and vinorelbine concentrations over a 72-h period following the start of pemetrexed administration in first cycle. In addition, blood samples were also collected from patients on days 0, 1, 2, 3, 4, 8 and 15 of the first cycle and days 0, 8 and 15 of the second cycle for measurement of TdR, dUrd, Hcy and MMA concentrations. Whole blood was collected into prechilled heparinised blood tubes and placed on ice immediately. Plasma was rapidly separated from cellular components by centrifuging at 3300 **g** at 4°C for 15 min. Plasma was then stored at −80°C and thawed just prior to analysis.

### Pharmacokinetic analysis

Plasma pemetrexed concentrations were analysed at Taylor Technology Inc. (Princeton, NJ, USA) using a validated liquid chromatography electrospray ionisation with tandem mass spectrometric detection method over the concentration ranges of 10–2000 and 1000–200 000 ng ml^−1^ (Latz *et al*, 2006). Samples were analysed for vinorelbine using a validated HPLC-fluorescence detection method over the concentration range of 2–500 ng ml^−1^ by Bioanalytical Laboratory Service (PPD Development, Richmond, VA, USA).

### Measurement of plasma TdR, dUrd, Hcy and MMA levels

Plasma TdR and dUrd levels were analysed using a validated LC-MS method, which we previously published (Li *et al*, 2003, 2005). Briefly, plasma samples were extracted with strong anion-exchange solid-phase extraction columns followed by HPLC separation and atmospheric pressure chemical ionisation mass spectrometry detection in a selected-ion monitoring mode. Plasma Hcy and MMA were analysed by Douglas Hanley Moir Laboratory (Sonic Clinical Trial Centre, North Ryde, NSW, Australia).

### Statistical analysis

PK parameters were evaluated by compartmental and multiple compartmental methods for pemetrexed and vinorelbine, respectively (Lee and Amidon, 1996). The area under the plasma concentration–time curve (AUC) was determined using the linear trapezoidal method with extrapolation to infinity. Total plasma clearance values of pemetrexed and vinorelbine for each patient were calculated by dividing the dose administered by the AUC. The values for plasma nucleosides, Hcy and methylmalonic acid at each time point are expressed as mean±s.d. Statistical analysis of data was achieved by Student's *t*-tests (paired) and regression correlation (GraphPad Prism). *P*<0.05 was considered to be statistically significant.

## RESULTS

### Pharmacokinetic analysis

The full PK for the phase I/II trial have been published previously (Clarke *et al*, 2005). The PK parameters for pemetrexed and vinorelbine for the subgroup of patients included in this study are summarised in Tables 2 and 3, respectively.

### Plasma TdR, dUrd, Hcy and MMA analysis

**Figure 2:**
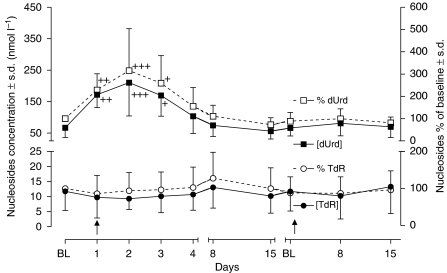
Plasma nucleoside concentrations and % of baseline changes following pemetrexed–vinorelbine treatment. Data are means±s.d. ^+^*P*<0.05, ^++^*P*<0.01 and ^+++^*P*<0.001 when compared with corresponding baseline values.

Plasma dUrd and TdR concentrations during the first and second cycles of treatment are shown in Figure 2. Plasma dUrd concentrations were significantly elevated on days 1, 2 and 3 after treatment at all pemetrexed dose levels. However, there was no significant change of plasma TdR following pemetrexed treatment. Mean pretreatment plasma dUrd and TdR concentrations of all 18 patients were 66.9±30.8 and 11.7±6.36 nmol l^−1^, respectively. Plasma dUrd in treated patients was significantly increased compared to baseline on days 1 (172 nmol l^−1^, 255%, *P*<0.01), 2 (210 nmol l^−1^, 315%, *P*<0.001) and 3 (170 nmol l^−1^, 252%, *P*<0.05) when averaged for all doses. By day 4, the plasma dUrd levels remained elevated relative to baseline, although this did not reach statistical significance (103 nmol l^−1^, 154%, NS). Deoxyuridine concentrations returned to baseline by day 8. In contrast, mean plasma TdR concentrations were 86, 94 and 96% of baseline on days 1, 2 and 3, and this was not significantly different to baseline values.

**Figure 3:**
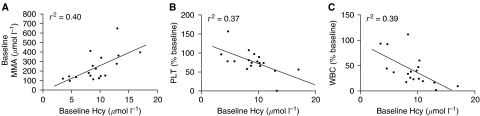
Correlation between baseline Hcy and (**A**) baseline MMA (*r*^2^=0.40, *P*<0.01), (**B**) PLT (*r*^2^=0.37, *P*<0.01) and (**C**) WBC (*r*^2^=0.39, *P*<0.01) on day 8 of treatment cycle 1.

**Figure 4:**
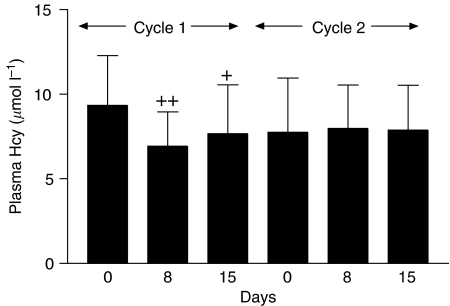
Plasma Hcy concentrations in treatment cycles 1 and 2. Data are means±s.d. ^+^*P*<0.05 and ^++^*P*<0.005 when compared with baseline concentrations.

The basal Hcy and MMA concentrations were 9.3±2.95 *μ*mol l^−1^ (range: 4.72–17.2) and 224±106 nmol l^−1^ (range: 97–412), respectively for the 18 patients. Most of the patients (17 out of 18) had Hcy values within the normal range (5–13.9 *μ*mol l^−1^), and only one patient had elevated Hcy (17.2 *μ*mol l^−1^). Five patients (5 out of 18) had abnormally high MMA levels and these ranged from 340 to 412 nmol l^−1^ (normal range: 73–271 nmol l^−1^). Baseline Hcy was significantly correlated with baseline MMA (*r*^2^=0.40, *P*<0.01), but only baseline Hcy correlated with the reduction (% baseline) of both platelets (*r*^2^=0.37, *P*<0.01) and leucocytes (*r*^2^=0.39, *P*<0.01) on day 8 of treatment cycle 1 (Figure 3). There was a significant reduction in plasma Hcy on days 8 (*P*<0.005) and 15 (*P*<0.05) compared to baseline values as shown in Figure 4. There was also a trend towards reduction in MMA levels after treatment; however, this did not achieve statistical significance. There was no correlation between either baseline Hcy or MMA concentrations with the maximum elevation of plasma dUrd in treatment cycle 1.

**Figure 5:**
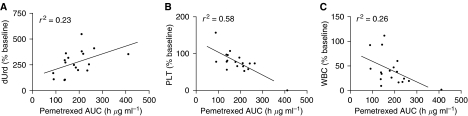
Correlation between total body exposure of pemetrexed (AUC) and (**A**) dUrd on day 2 (*r*^2^=0.23, *P*<0.05), (**B**) PLT (*r*^2^=0.58, *P*<0.001) and (**C**) WBC (*r*^2^=0.26, *P*<0.05) on day 8 of treatment cycle 1.

The total body exposure of pemetrexed (AUC) was significantly correlated with reduction (% baseline) of platelets (*r*^2^=0.58, *P*<0.001) and leucocytes (*r*^2^=0.26, *P*<0.05) on day 8 and maximum increase (% baseline) of dUrd (*r*^2^=0.23, *P*<0.05) on day 2 as shown in Figure 5. However, there was no significant correlation between the change in plasma dUrd and platelet and leucocyte counts on either day 8 or 15.

## DISCUSSION

The objective of this study was to investigate PK–PD aspects of pemetrexed in the setting of a phase I/II combination study. The PK of pemetrexed and vinorelbine were consistent with data obtained with single-agent chemotherapy (Marquet *et al*, 1992; Leveque and Jehl, 1996; Sabot *et al*, 1998). There was no evidence for PK interaction between the two drugs. In the current study, measurement of plasma dUrd concentration showed that pemetrexed administered at doses of 400–700 mg m^−2^ produced consistent elevations in plasma dUrd, suggesting that plasma exposure to pemetrexed was sufficient to induce TS inhibition in patients. Indeed, the current results have demonstrated a clear PK–PD relationship for TS inhibition in these patients (Figure 5). On days 1–3, plasma dUrd levels were significantly higher than baseline, and this may suggest rapid TS inhibition after dosing. The maximum increase of dUrd level was observed on day 2 following which plasma dUrd gradually returned to baseline by day 8, suggesting that TS inhibition was short-lived with this schedule of pemetrexed. This pattern is comparable to previous studies with the classical anti-folate raltitrexed (RTX), which showed dUrd elevations on days 1–3 and which returned back to baseline within 2 weeks of treatment (Ford *et al*, 2002; Horton *et al*, 2005).

Intracellularly, pemetrexed is polyglutamated by a reaction catalysed by folylpolyglutamate synthase. Polyglutamated pemetrexed is approximately 60-fold more potent in its inhibition of TS than the parent monoglutamate compound (Shih *et al*, 1998) and is retained intracellularly long term. Therefore, the highly inhibitory polyglutamated forms would be expected to generate long-lived TS inhibition. However, similar to the TS-selective inhibitor RTX, TS inhibition by pemetrexed, as indicated by elevations in plasma dUrd concentrations, was relatively transient. There are a number of possible explanations for this. Firstly, plasma dUrd concentrations may not be sensitive indicators of TS inhibition. However, in a previous study of capecitabine we saw protracted elevation of plasma dUrd concentrations that lasted for the entire course of treatment (Li *et al*, 2007). An alternative explanation is that inhibition of TS by folate-based agents is not durable, in spite of intracellular polyglutamation, and can be competed out by naturally occurring folates once plasma drug levels are reduced. This competition would be enhanced by exogenous administration of folic acid, which could explain why vitamin-supplemented patients do not experience protracted, potentially lethal toxicity.

Regardless of the explanation, these data suggest that other more frequent administration schedules of pemetrexed, such as bi-weekly regimens, should be explored to optimise its antiproliferative effects. Although cell death produced by pemetrexed can be prevented in culture through the addition of exogenous TdR, the plasma concentration of TdR observed in patients is likely to be too low to play a significant role in the modulation of pemetrexed activity through salvage pathways. Therefore, human plasma TdR level does not appear to be a useful PD phenotypic marker for TS inhibition.

Because anti-folates can interfere with the normal metabolic pathway of folates in the body, it might be reasonable to speculate that treatment with pemetrexed would alter folate metabolism and that this would be reflected by an elevation of plasma Hcy concentration. Surprisingly, the current study has shown that plasma Hcy was not elevated but instead decreased in the first 2 weeks following pemetrexed therapy (Figure 4). Indeed, it has been previously reported that plasma Hcy concentrations are not affected by pemetrexed (Niyikiza *et al*, 2002). However, patients did not receive folate supplementation in that particular study. Therefore, it is likely that the kinetics of reduction of plasma Hcy observed in current study are the direct result of expansion of intracellular folate pools by folate/B_12_ supplementation. This is consistent with previous studies that have demonstrated that folate supplementation, with or without additional B_12_ and B_6_, can significantly lower plasma Hcy (Naurath *et al*, 1995; Refsum *et al*, 1996; Bronstrup *et al*, 1998; Homocysteine Lowering Trialists' Collaboration, 1998; Bunn *et al*, 2001; Billion *et al*, 2002).

In the present study, a statistically significant relationship between baseline Hcy and MMA was established in patients. This correlation is consistent with similar observations in healthy volunteers as well as a number of patient subset studies, although MMA is usually indicative of cobalamin status whereas Hcy is the preferred marker for folate deficiency (Valik *et al*, 2004). Indeed, only baseline Hcy was statistically associated with increased risk of neutropenia and thrombocytopenia on day 8 of treatment cycle 1 (Figure 3). This supports the notion that plasma Hcy is a more specific marker for overall functional folate status and indicates that baseline Hcy might be useful in predicting toxicity during pemetrexed therapy or be used as support for increased supplementation (either duration or dose) in individuals in whom Hcy remains elevated after initial supplementation. In addition, it also supports the current use of up-front supplementation with folic acid and B_12_ in patients prior to treatment with pemetrexed.

In summary, the results of this study demonstrate PD changes consistent with TS inhibition and a close PK–PD relationship following pemetrexed–vinorelbine treatment. Baseline Hcy appears to be a better predictive marker than MMA for haematological toxicity. The current results also support the use of vitamin supplementation to ensure normal vitamin B_12_ and folate status to control haematologic toxicity secondary to pemetrexed administration.

## Figures and Tables

**Figure 1:**
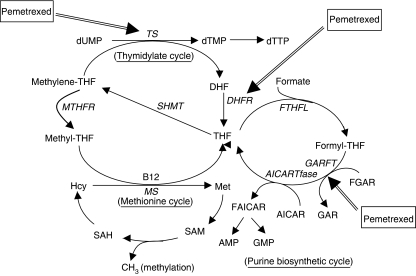
Folate involved in biosynthesis of pyrimidine, purine and methionine. AICARTfase, aminoimidazole carboxamide ribonucleotide transformylase; AMP, adenosine monophosphate; GMP, guanosine monophosphate; FAICAR, formylaminoimidazole ribonucleotide; FGAR, formylglycinamide ribonucleotide; FTHFL, formate-tetrahydrofolate ligase; MTHFR, methyltetrahydrofolate reductase; SAM, *S*-adenosylmethionine; SAH, *S*-adenosylhomocysteine; SHMT, serine hydroxymethyltransferase.

**Table 1:** Patient demographics and clinical characteristics (*n*=18)

**Parameter**	**No. of patients (%)**
Male	13 (72%)
Median age, years (range)	58 (40–72)
	
*Performance status*	
0	5 (28%)
1	13 (72%)
	
*Disease status*	
Metastatic	17 (94%)
Locally advanced	1 (6%)
	
Prior adjuvant chemotherapy	0
Prior chemotherapy for metastatic disease	12 (67%)
Prior chemotherapy for locally advanced disease	1 (6%)
Prior radiotherapy	5 (27%)

**Table 2:** Summary of PK parameters for pemetrexed

	**Pemetrexed expressed as mean±s.d.**			
Dose (mg m^−2^)	400	500	600	700
No. of patients	6	5	3	4
AUC_0–*∞*_ (h *μ*g ml^−1^)	130±36.4	194±75.8	187±30.5	216±29.3
*C*_max_ (*μ*g ml^−1^)	89.1±25.2	134±29.4	105±10.5	131±18.4
CL_p_ (l h^−1^ m^−2^)	3.29±0.96	2.59±0.94	3.28±0.58	3.32±0.48
*t*_(1/2)_ (h)	2.57±1.13	2.58±1.47	1.91±0.49	3.00±0.85
*V*_ss_ (l m^−2^)	10.3±3.71	7.19±1.68	8.07±1.41	10.9±2.87

AUC=area under the concentration–time curve; CL_p_=plasma clearance; *C*_max_=maximum plasma concentration; PK=pharmacokinetic; s.d.=standard deviation; *t*_(1/2)_=terminal elimination half-life; *V*_ss_=steady-state volume distribution.

**Table 3:** Summary of PK parameters for vinorelbine

	**Vinorelbine expressed as mean±s.d.**		
Dose (mg m^−2^)	15	23	30
No. of patients	7	3	8
AUC_0–*∞*_ (h *μ*g ml^−1^)	0.88±0.60	1.78±0.38	2.04±1.03
*C*_max_ (*μ*g ml^−1^)	1.48±1.13	2.91±0.14	2.98±1.83
CL_p_ (l h^−1^ m^−2^)	27.4±22.9	12.9±0.62	18.7±9.73
*t*_(1/2)_ (h)	48.3±15.2	36.5±8.21	42.0±11.6
*V*_ss_ (l m^−2^)	1067±543	1671±373	1649±1240

AUC=area under the concentration–time curve; CL_p_=plasma clearance; *C*_max_=maximum plasma concentration; PK=pharmacokinetic; s.d.=standard deviation; *t*_(1/2)_=terminal elimination half-life; *V*_ss_=steady-state volume distribution.
